# An application of analytic network process model in supporting decision making to address pharmaceutical shortage

**DOI:** 10.1186/s12913-020-05477-y

**Published:** 2020-07-08

**Authors:** Leila Zarei, Najmeh Moradi, Farzad Peiravian, Gholamhosein Mehralian

**Affiliations:** 1 Pharmacoeconomics and Pharma Management, Health Policy Research Center, Institute of Health, Shiraz University of Medical Sciences, Shiraz, Iran; 2 Pharmacoeconomics and Pharma Management, Health Management and Economics Research Centre, Iran University of Medical Sciences, Tehran, Iran; 3 Pharmacoeconomics and Pharma Management, School of Pharmacy, Shahid Beheshti University of MedicalSciences, Tehran, Iran

**Keywords:** Equity, Efficiency, Effectiveness, Resource allocation, Scarce drugs allocation, Need-based resource allocation, Analytic network process

## Abstract

**Background:**

The present study aimed to develop an Analytic Network Process (ANP) model to assist policymakers in identifying and prioritizing allocation indicators, which are being used or should be used to distribute drugs in short supply among different provinces.

**Methods:**

The model encompasses the interactions between various indicators and efficiency, equity, and effectiveness paradigms. Accordingly, a set of clusters and elements, which were associated with the allocation of drugs in short supply in Iran’s pharmaceutical system, were detected to develop the model and were then compared in pairs in terms of a specified factor to show the priorities.

**Results:**

Equity had the highest priority (0.459) following by Efficiency (0.37), and Effectiveness (0.171). The 4 most important allocation indicator were “number of prescriptions” (0.26) and “total bed occupancy rate” (0.19) related to equity, “total population” (0.21) in efficiency and “the burden of rare and incurable disease” (0.07) in effectiveness paradigm.

**Conclusions:**

The capability to overcome inefficient resource allocation patterns caused by both oversupply and undersupply derived from historic resource allocation may be highly limited in the absence of the need indicators. The quality of the decision is related to a careful balancing act of the three paradigms which represents roughly the triple aim of public healthcare systems: clinical improvement (effectiveness), population health improvement (equity and access), and reducing cost (economic aspects -efficiency).

## Background

Drug shortage occurs because of a failure to follow predetermined supply programs [1]. At the time of shortage, the health systems’ immediate action is essential to prevent disruptions in patient care [2]; hence, activities associated with drug shortage management need to be of priority, compared to other tasks, to ensure the supply of an essential medication or its alternative [2, 3]. A number of studies point out that drug shortage has been increasing over the recent decades [3, 4], posing the health care systems an ongoing challenge [2]. Furthermore, there is no unified theory or framework defined to manage such issues [2, 5].

Currently, Iran’s pharmaceutical system is suffering from regular short supply of some medications, especially those used to treat non-communicable diseases [6], in the market, and the inefficient structure of the current supply chain has also aggravated the problem [7, 8]. Although there has been a significant increase in the number of individuals having access to basic pharmaceuticals over the past decade, the lack of access to sustainable resources is still a problem [9]. However, increasing the accessibility of medications in short supply is not possible unless a reliable drug supply system, which can ensure an adequate distribution of resources, is operating [10].

A review of the experiences of other countries reveals a need to shift the discussion from questioning to implementation of best practices in this field [11]. Employing a number of mechanisms, categorized in the form of supply and demand side mechanisms, to allocate rare healthcare resources is possible [12]. There are different indicators in each side, and a simple/ a single indicator as well as composite indicators has been utilized by different countries to weigh the population of the district. In several countries concerned with the concept of needs in the allocation of their resources, composite indicators of socioeconomic conditions, including deprivation and asset indicators, have been employed. Demographic and socioeconomic status, including population size, age, gender, and health status are also commonly applied in developing countries [13–16].

### Challenges of Iran’s pharmaceutical system regarding drugs in short supply

In Iran, Iran Food and Drug Administration (IFDA) is in charge of supervising all issues posed in the pharmaceutical sector. Ensuring public access to effective and safe medications at affordable prices, specifically those stipulated in the Islamic Republic of Iran’s 20-year national vision document and Iran National Drug Policy (INDP) [17, 18], is one of the primary goals of IFDA.

Since drug shortage is inevitable, policy makers should be provided with dependable and appropriate information to offer successful and efficient management for the problem [2, 5, 19]. According to some practical evidence, IFDA implements a proactive inventory program to collection thorough information about drugs in short [9]. IFDA’s information center of medicine, universities of medical sciences, and some referral pharmacies across the country gather the required information. Despite collecting data via routine market monitoring across the country, the warning system cannot be considered as a comprehensive system and should be upgraded to an advanced structure through enhancing collaboration among stakeholders and improving IFDA’s capability to exert control over the pharmaceutical market [7]. Another responsibility of IFDA is to ensure that a definite distribution policy is in place during shortages. Evidence has revealed that provinces are prioritized when drug shortages occur in the country [1]; therefore, this controlled allocation program can be regarded as a type of implicit healthcare rationing. Like other countries, it is proposed in Iran that clear policies governing the allocation of scarce resources help the healthcare system to allocate this valuable resource successfully [20], and that some considerations are needed to determine factors leading to variations among the populations of each province in order to perform rationing effectively [21, 22].

### Need-based resource allocation

According to the World Health Report 2000, the goals of health systems are good health for citizens, accountability for what the population expects, and equitable means of funding operations. Accordingly, a health care system is mostly evaluated by its progress towards the goals.

Duckett (2008) suggested a two dimensional method to evaluate the health care systems, with the first dimension addressing quality, efficiency as well as acceptability and the second dimension addressing equity [23]. Furthermore, some studies have discussed conflicts and trade-offs among different evaluation measures of the health systems, including effectiveness, efficiency, and equity [24]. Berwick et al. (2008) argued improving the health care system requires simultaneous pursuit of three aims: improving the experience of care, improving the health of populations, and reducing per capita costs of health care [25].

On the other hands, all health systems struggle with the issue of meeting population health needs fairly under resource constraints [26]. Equity issues in resource allocation, especially in rationing the scarce drugs should reduce health inequalities by adopting some policies, particularly the ones made to minimize or mitigate the effects of the unequal distribution of resources affecting individuals’ health status [27]. But the issue raised here goes beyond equity. The ethical aspects of such decisions has been discussed in the accountability for reasonableness framework (A4R) of Norman Daniels [26]. Theoretically, the allocation problems pose formidable challenges to individuals who have to decide on the allocation of health care resources at the times of scarcity [28, 29]. A key recommendation for meeting the A4R is that establishing a fair process for priority setting is easier than agreeing on principles [26]. Norman Daniels (1982) examined the ethical presuppositions and implications of three accounts of equitable access, a “use- (or use-per-need)” based account, a “process” variable account, and “market” account [30] with an ethical balancing.

More recently, academics have suggested a number of factors as the ones probably associated with need-based resource allocation to direct such decision processes. As McIntyre and Anselmi (2012) stated, the most prevalent indicators are demographical make-up, socio-economic conditions, ill-health levels as well as population size [16]. Obviously, in the absence of any gold standard measure for a population’s health care need, the selection of valid, dependable, and accountable indicators (or proxies) of health care is of great importance [21, 31]. Needless to note, when rationing decisions are made, careful weighing of the indicators is of paramount importance to make sure that the indicators are not “irrational” or “ethically objectionable as when they reflect discriminatory attitudes” [20].

### ANP and fuzzy ANP

Since the 1970s when Saaty proposed the Analytic Hierarchy Process (AHP), its application has become prevalent in the Multiple Criteria Decision-Making (MCDM) environment to address sophisticated decision-making problems [32, 33]. ANP, a generalized form of AHP, can be applied as an efficient tool in the cases where the interactions among the elements lead to the formation of a network structure being beneficial under different real-world conditions [34]. ANP approach is widely used for prioritization, performance evaluation, and other contexts [35, 36], and it can detect feedback and interdependent associations among and between the components [37].

Although ANP aims at capturing the expert’s knowledge, its traditional version failed to consider the human thinking style, and, consequently, a fuzzy ANP (FANP) was proposed. The application of fuzzy theory in decision-making problems has provided favorable practical results [38]. Given that interval judgments is usually more convenient for the decision-makers than the fixed-value judgments, the application of a fuzzy ANP is valid even in the cases of unavailable information or costly dependable information [39]. The difference between two ANP methods lies in extracting the weights of the pairwise matrix; otherwise, they are the same. Several researchers have utilized a fuzzy ANP-based method to solve complicated decision-making scenarios [40–42]. Both of these methods have been applied in healthcare settings [36].

### ANP in pharmaceutical decision-making

On the one hand, decisions made in the pharmaceutical sector can have a direct impact on the living conditions and health of the community. On the other hand, improving health standards can provide the grounds for economic development [43]. The pharmaceutical sector should consider a large number of factors, particularly in resource allocation process, to ensure the equitable distribution of resources and, ultimately, an optimal use of scarce resources [10]. Accordingly, the use of MCDM methods is particularly critical for pharmaceutical decision-makers to support their decisions. ANP can be defined as a multi-criteria theory, which enables the decision-makers to structure a decision in the greatest conceivable general manner. The representation of any decision problem with no concern about what comes first or next is possible using a network structure when the sources, cycles, and sinks are addressed [44].

Applying ANP as MCDM can provide a more data-driven and transparent decision process while taking intangible aspects and decision-makers’ preferences into account [44]. Furthermore, some recent studies have suggested that ANP can be used as a part of a benchmarking effort in selecting the best practice solution [45]. Feibert et al. (2016) applied ANP to evaluate pharmaceutical distribution solutions based on a set of decision criteria specified to a healthcare logistics context [36]. A managerial decision model was proposed by Machado et al. (2014) and considered a Portuguese pharmaceutical supply chain using ANP.

A thorough review of resource allocation in Iran’s pharmaceutical system reveals the lack of suitable allocation/distribution indicators; therefore, the detection of the relevant indicators would go a long way toward an adequate allocation of scarce pharmaceutical resources. To fill this gap, some further studies to evaluate the allocation process of scarce drugs and to determine the relevant indicators seems to be of greater urgency. The present study aimed to identify and prioritize the allocation indicators using an ANP model to assist policymakers in distributing scarce drugs among different provinces. The results of this study would contribute the existing literature to reduce the impact of drug shortages such as irreparable damage and premature death.

The contribution of this study lies in its potential to provide a deeper understanding of the factors to be considered by policymakers in order to decrease the negative impact of misdistribution/misallocation of scarce drugs through developing a conceptual model in accordance with ANP. The study also extends the existing literature on health-care resource allocation through determining the critical indicators for managing shortages in Iran based on the need-based resource allocation concept.

## Methodology

**Fig. 1:**
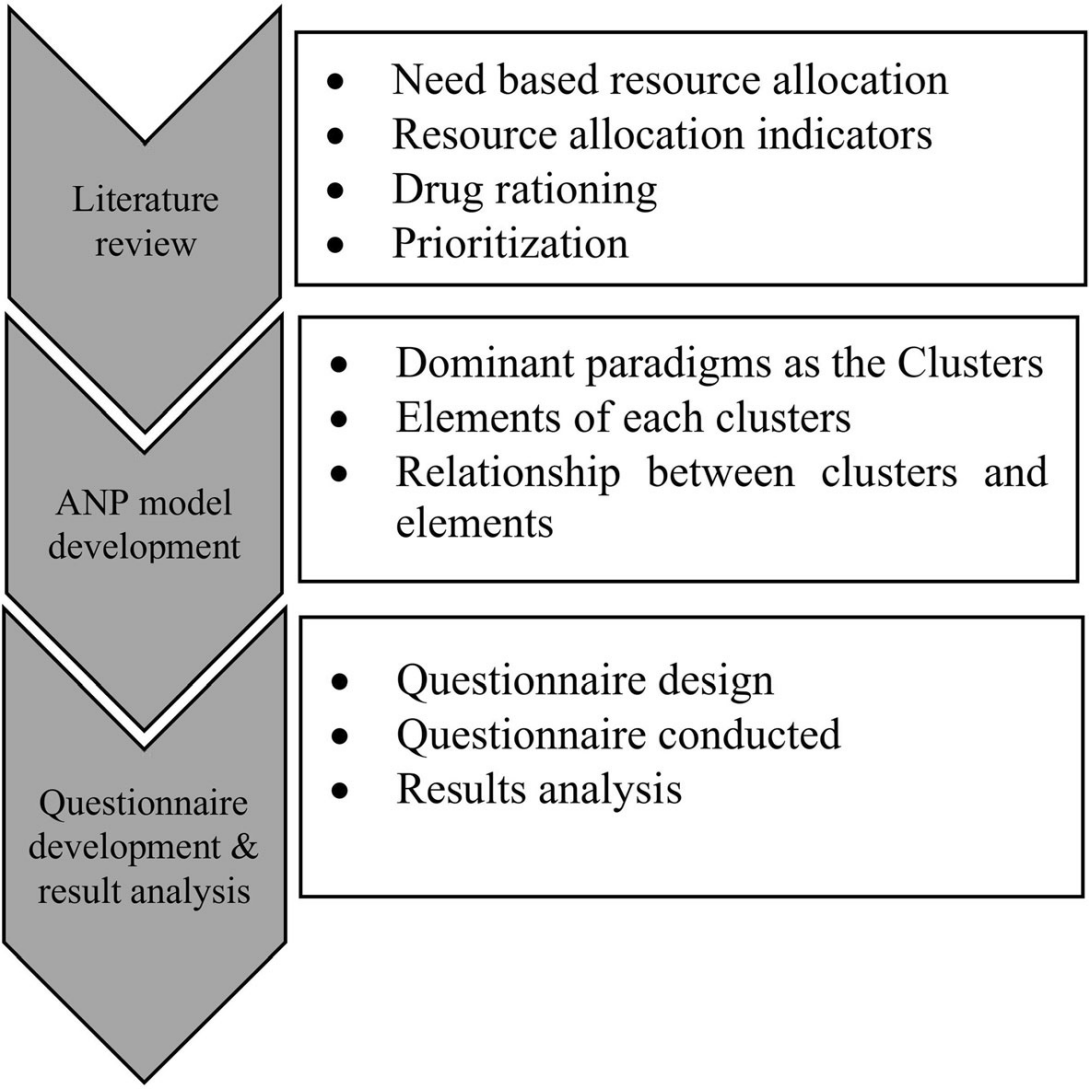
Methodology

Three consecutive steps were taken to conduct the present study. The methodology of this study is summarized in Fig. 1.

First, to choice the allocation indicators, three phases were conducted: The first was literature review to find what indicators employed in the priority setting decision context in health systems around the world. Then, in the second phase, the indicators scrutinized by fifty academics and executives who were specialists in pharmaceutical resource allocation and distribution (including the vice chancellors of food and drug in universities of medical sciences along with other specialists of the field). Finally, in third phase, a structured process was performed based on the Delphi technique requirements to finalize the indicators.

Furthermore, according to the expert consultation meetings, the indicators affecting the distribution of scarce drugs among different provinces in Iran are not completely separated and are interdependent as such the multi-criterion techniques such as ANP to consider interactions at the time of weighting is more convenient. Afterwards, the construction of ANP model was developed according to both review of previous studies and brainstorming decision-makers’ opinions. To run the pairwise comparisons between clusters/elements of ANP model, a questionnaire was developed and submitted to some experts. The phase was completed by 14 respondents, which 6 were academics and have pharmaceutical policy making experience, 4 were executive members who worked in Iran FDA particularly in time of crisis and shortages and 4 were specialists in pharmaceutical resource allocation. The questions were measured based on a nine-point scale, in which 1 represented equal significance and 9 indicated extra significance of one element compared to another one. Finally, the results were analyzed using the Super Decisions software version 2.2.

In this paper, we ranked allocation indicators for drugs in shortage using the fuzzy ANP technique. A variety of fuzzy ANP methods have been introduced by different researchers. In the present study, the extent analysis method was used [46]. The steps involved in fuzzy ANP are listed below [47]:

### Step 1. Establishment of the model and problem

The main goal is divided into sub-goals, criteria/sub-criteria and alternatives. Goal can be defined as what is supposed to be obtained. While a set of parameters on which decision depends is called criteria, alternatives represent the elements according to which decision-making should be performed.

### Step 2: establishment of a fuzzy pairwise comparison matrix (independent as well as interdependent) and Defuzzification

Criteria and alternatives are also defined as a scale based on the qualitative scale of significance proposed by Saaty [34] and are subsequently converted into the quantitative scale ranging from 1 to 9. Several forms of fuzzy numbers are available, although Triangular Fuzzy Numbers (TFN) and trapezoidal fuzzy numbers have the highest popularity [48]. Different trapezoidal/triangular fuzzy numbers could be classified in order through the portrayal of their curves in a fuzzy multiple-criteria decision-making problem. Unless its order can be ranked by its own figures, other approaches could be applied alternatively.

Pairwise comparison was run after scaling. This means that the corresponding significance of component *i* relative to component *j* with regard to the parent component in the hierarchy is identified and allocated to the position (i. j) of the pairwise comparison matrix [35]. The priorities among clusters, within cluster elements, and between various cluster elements were established based on pairwise comparisons as well as judgments. The local priority vectors (Eigenvectors) obtained from comparison matrix resulted in unweighted super-matrix when obtaining global priorities in a system with interdependent impacts. Then eigenvectors were entered into the suitable columns of a matrix according to the impact flow from a component to another component, i.e. a supermatrix. The supermatrix exhibits similarities to Markov chain process [49]. There are different available defuzzification methods, out of which Liou and Wang’s (1992) method was used in this paper, taken from the study of Wu et al. [49].

### Step 3: determination the eigenvectors and Supermatrix formation

The calculation of the corresponding significance is performed using eigenvalues along with eigenvector of the comparison matrix.

Given the usual interdependencies of the clusters across a network, the columns of a supermatrix typically contain values larger than one. The transformation of the supermatrix is first necessary to specify its stochastic properties, indicating that each column of the matrix enhance the unity. The approach recommended by Saaty (1996) includes determining the corresponding significance of the clusters in the supermatrix, in which the column cluster (block) represents the controlling component. This means that the comparison of the row components having blocks with non-zero entries in the same column block is performed based on their impacts on the components of the same column block [49]. An eigenvector is to be acquired for each column block using the pairwise comparison matrix of the row components with regard to the column component. For each column block, the first entry of the corresponding eigenvector is multiplied by all the elements in the first block of the same column. The same process is carried out for the next column blocks, according to which the blocks in each column of the supermatrix are weighed, resulting in a weighted supermatrix with stochastic features. Increasing a matrix power would better reflect the long-term corresponding impacts of the elements on each other. To obtain the convergence of the significance weights, the weighted supermatrix is raised by the power 2 k + 1, where k indicates a randomly large number, and the new matrix is known as the limit supermatrix [50]. The same procedure is performed for each level of the hierarchy as long as the decision-making process is completed. In the case of similar entries for each column, the limit matrix is achieved and the matrix multiplication process continues no further. The normalization of the blocks in this supermatrix results in eventual priorities for all the elements of the matrix.

### Step 4: evaluation of the decision

If the supermatrix obtained in the previous step covers the entire network, it is possible to find the priority of weights for the alternatives in the columns of the normalized supermatrix. On the other hand, when a supermatrix just includes interrelated components, further calculation is needed to achieve the total priorities for the alternatives. The alternative with the highest total priority will be selected.

It is of great importance to make consistent comparisons to ensure that judgment reliability is considered. To this end, Consistency Index (CI) and Consistency Ratio (CR) are proposed. CR and CI represent the consistency ratio and the consistency index of the random reciprocal matrix produced by a quantitative 9-point scale [32]. If CR < 0.1, it is accepted; otherwise, the pairwise comparison requires revision.

## Results

After reviewing the methods and effective indicators of scarce resources allocation in the world, about 20 indicators was detected, which based on availability of data in Iran, 16 indicators entered to the next step. Then, in the second phase, the indicators scrutinized by experts and just 3 indicators were rejected and 13 indicators entered to the Delphi phase. Finally, the Delphi technique was performed and 8 indicators contained the burden of endemic, special, rare and incurable and traumatic diseases, total population, the population of non-resident patients, total bed occupancy rate, number of prescriptions and the number of general practitioner (GPs) and specialist were approved. Our finding showed that eight of the common measures/indicators of resource allocation were rated face valid for measuring need and currently used in need-based resource allocation formula in other countries. (for further information, see Zarei (2019)) [51]. According to Berwick (2008) [25] and Wenzel (2008) [24], clinical improvement (effectiveness), population health improvement (equity and access), and reducing cost (economic & resources aspects -efficiency) are the paradigms for the allocation of limited resources or explicit priority settings. The results of this section are presented in Table 1.

**Table 1:** Paradigms and indicators of scarce drug allocation

Clusters/ Paradigms	Paradigms definition	Paradigms elements/indicator	Indicator definition/ Operationalization
Efficiency	The ability to avoid wasting resources in doing something or in producing a desired result. In a more general sense, it is not possible to make anyone better off without making someone else worse off.	Total population	Population size in a geographical area.
		Non-resident patient	Patients are not resident in the place of receipt of health service.
Equity and Access	A mix of equal inputs for equal need and equal access for equal need.	Number of health professionals	Number of general practitioner and specialists working in each province.
		Total bed occupancy rate	The number of beds effectively occupied (bed-days) of each province.
		Number of prescriptions	The total number of prescriptions filled annually in each province.
Effectiveness	What extend a goal could be reached. If a goal cannot be reached, any resource input is wasted. The measure of effectiveness can be multidimensional.	Burden of diseases	**Burden of endemic diseases:** The burden of the diseases that always present in a certain population or region.
			**Burden of special, rare and incurable diseases:** In Iran, dialysis patients, thalassemia, hemophilia, cancer, MS, kidney transplant, diabetes and E. B are covered by this group.
			**Burden of traumatic diseases:** Any injury that goes beyond the body’s resilience and leads to lesions in the body is called trauma, which includes accidents, events, falls from heights, and even the psychological and chemical damage.

Next, a network of indicators affecting scarce drug allocation was developed and prioritized using ANP method. The computational procedure of ANP method is summarized below:

### Step 1. Establishment of the model and problem

**Fig. 2:**
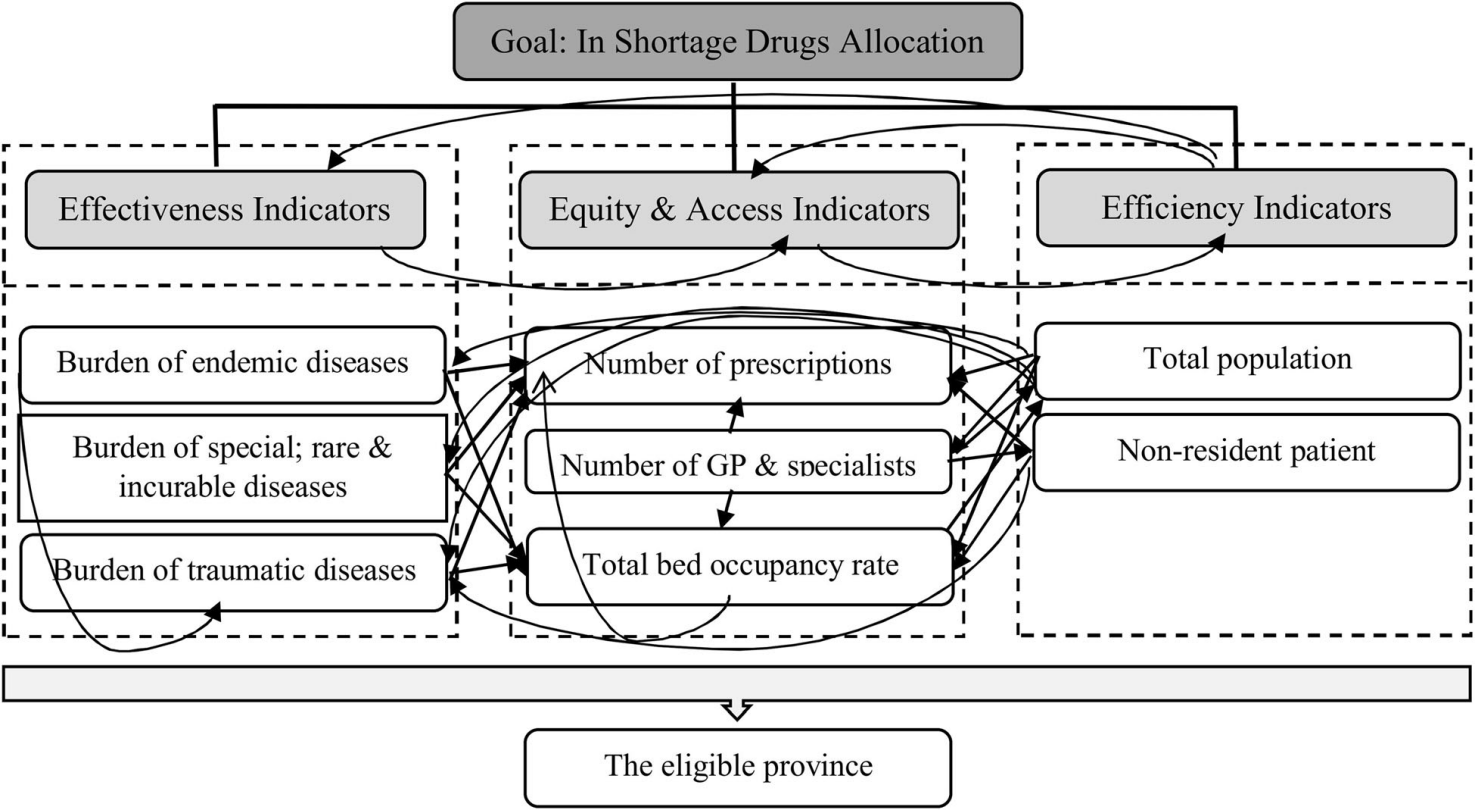
The ANP model

The ANP model (Fig. 2) shows the inner and outer dependencies. The model is supposed to determine the most important indicators of optimal allocation/distribution of drugs in shortage.

As presented in Fig. 2, a unidirectional arrow shows that one cluster/cluster element has impacts on the other ones (outer influence or inner influence). This arrow goes from the “Efficiency” cluster toward the “Equity and access” cluster, indicating that the value of the “Equity and access” cluster depends on the judgments made in the “Efficiency” cluster (see Appendix Table S1).

A 45-item questionnaire was developed with regard to the relationship determined by ANP model and submitted to 14 experts to elicit the most important indicators for the allocation/distribution of drugs in the short supply (The questionnaire are presented in Appendix 2). A response rate of 89% was obtained. Pairwise comparisons were performed using a nine-point priority scale with 1 showing identical significance and 9 representing the extreme significance of an element compared to the other’s.

### Step 2: establishment of the fuzzy pair-wise comparison matrix (independent and interdependent) along with Defuzzification

Although different forms of fuzzy numbers could be used, we applied triangular fuzzy numbers (TFN) in this study. TFN is simply represented as (L, M, U), where the parameters L, M, and U indicate the smallest probable value, the most promising value, and the biggest probable value in describing a fuzzy event, respectively. TFN can be applied to indicate the fuzzy opinions as well as the expert consensus. In this paper, we used the procedure suggested in Wu’s et al. (2008) [49] study to define TFN. In Table 2, the fuzzy aggregate pair-wise comparison of the decision paradigms is presented with regard to the main goal. After defuzzification, the fuzzy weight of paradigms was converted into a certain weight and was the normalized.

**Table 2:** Fuzzy Aggregate Pair-wise Comparison and weight of the paradigms

Criteria/Paradigms	Efficiency	Equity & Access	Effectiveness	Normal weight	Certain weight
**Efficiency**	(1,1,1)	(0.38,0.449,0.551)	(0.302,0.35,0.405)	0.166	0.168
**Equity & Access**	(1.815,2.228,2.634)	(1,1,1)	(1.063,1.197,1.351)	0.424	0.428
**Effectiveness**	(2.472,2.853,3.315)	(0.74,0.835,0.941)	(1,1,1)	0.409	0.413

CR^m^:0.021 consistency ratio (mean values); CR^g^:0.063 consistency ratio (geometric means of lower and upper bounds)

Afterwards, these steps were repeated for different sub-criteria with respect to the relevant paradigms, and there was a pair-wise comparison of the internal relationships between each cluster/paradigm and sub-criteria (elements).

### Step 3: determining the eigenvectors and Supermatrix formation

After measuring the preferences using the Improved Fuzzy AHP (IFAHP) method, the final weights of the criteria and sub-criteria were achieved (see Appendix Table S2).

The Appendix Table S3 shows the supermatrix along with corresponsing vectors and matrices, which were previously achieved. Since the supermatrix consists of the interactions between clusters, inner dependence is present among criteria and witnin sub-criteria, and the columns contained no 1. The transformation of a weighted supermatrix was first performed to make it stochastic (see Appendix Table S4). Following the insersion of the normalized values into the supermatrix and the completeion of the column stochastic, the supermatrix power was enhanced to an acceptable level so that convergence was observed. Convergence was reached for the current supermatrix, and a unique eigenvector was also obtained. The Appendix Table S5 indicates the final limit matrix, which is column-stochastic and reflects the final eigenvector. Finally, the final and normalized weight as well as the priority of each criterion/element was obtained (Table 3).

**Table 3:** The final weight and priority

Clusters/ Paradigms	Elements	Weights of elements in their clusters	Priorities of elements	Weight of elements in overall	Weights of clusters	Priorities of clusters
Efficiency^a^	Total population	0.694	2	0.211	0.370	2
	Non-resident patient	0.306	5	0.075		
Equity and Access^b^	Number of general practitioner and specialists	0.172	7	0.046	0.459	1
	Total bed occupancy rate	0.383	3	0.190		
	Number of prescription	0.445	1	0.262		
Effectiveness^c^	Burden of endemic diseases	0.341	8	0.053	0.171	3
	Burden of special, rare and incurable diseases	0.375	4	0.077		
	Burden of special, rare and incurable diseases	0.284	6	0.068		

^a^CRm: 0.011، CRg: 0.056, ^b^ CRm: 0.014، CRg: 0.035, ^c^CRm: 0.028، CRg: 0.074

The priority synthesis with respect to the goal was as follows: Equity and Access (0.459), Efficiency (0.37), and Effectiveness (0.171).

According to the model results, the most important indicators are ‘number of prescriptions’ and ‘total bed occupancy rate’ if the equity paradigm is considered and ‘total population’ if the efficacy paradigm is considered. According to the results of the limit matrix, ‘number of prescriptions’ (W = 0.262) and ‘burden of endemic diseases’ (W = 0.053) were the most and the least important indicators for allocation of scarce drugs, respectively. Furthermore, total population is introduced as the second most important indicator, followed by ‘total bed occupancy rate’.

Moreover, “burden of special, rare, and incurable diseases” (W = 0.077) in the cluster “Effectiveness” had the highest weight and was the main indicator in the cluster.

### Step 4: evaluation of the decision

All the clusters underwent the consistency analysis, resulted in a consistency ratio < 0.1, which always presented and reflected consistent results.

## Discussion

Health resources allocation processes differ across countries and fundamentally depend on how the delivery of healthcare and related services is organized. In Iran, a limited ration of pharmaceutical products was distributed by IFDA in the case of scarcity; indicating that IFDA decides how best to ration allocations. This study aimed to identify and prioritize scarce drug allocation indicators to assist Iranian healthcare policymakers in making more efficient, effective, and equitable decisions.

According to the results of previous studies and the review of the relevant literature, eight need-based indicators of scarce drug allocation were detected and classified into three clusters, namely efficiency, equity, and effectiveness. This classification had been previously used by some other researchers [24, 52–54]. For instance, Berwick et al. (2008) claimed the triple aim of public healthcare systems are clinical improvement (effectiveness), population health improvement (equity and access), and reducing cost (economic aspects -efficiency) [25]. The results from ANP technique suggest that “equity and access” is the most important paradigm for the allocation of scarce drugs. This finding is consistent with those of the other studies [27, 55–57]. Because based on the current situation, when a drug is in short supply in a province, it is possible that the neighboring province, which does not need it, will be in abundance. So, after sharing information, some provinces may feel discriminated against. This highlights the importance of the equity aspect.

In terms of access, the problems associated with sanctions and the provision of Active Pharmaceutical Ingredient (API) have always bothered our country, and drug suppliers have always faced the problem of access, Iran has been under in shortages since 2012 due to sanctions. This is more even challengeable in the case of access to medicine for incurable and rare disease. In the case of burden of rare disease, due to their heavy social impacts, participants have always been considered because of their responsiveness to these patients and the specific political burden that these patients can place on the system.

In addition, “number of prescriptions” is the most important indicator in this cluster. In other words, “number of prescriptions” is the most acceptable indicator among all the studied indicators, implying the significance of including the needs in the supply side. In the absence of adequate direct need-based data, it would be difficult to distinguish the effects of true needs on utilization from the effects of supply and demand. Gaminde (1999) [58] argues that the effects of supply side (by the prescribers) in pharmaceutical sector is greater than those of the demand side (by the consumers). Accordingly, the number of prescriptions as a proxy of need determined by the prescribers plays a critical role in the allocation of scarce drugs. This finding is also in a similar vein with the findings of the previous studies [59].

The first indicator of need is population size in a geographical area [13] as such an increase in the covered population is expected to enhance the pharmaceutical resource use due to the increased utilization of health services [14]. Like the findings in [13, 21, 31, 60], “total population” in this study was the most important indicator in the efficiency cluster. In addition, “total bed occupancy rate” as another indicator in the equity cluster was also listed as the first three important indicators. This finding is also similar to the findings in [57, 59].

Moreover, the results of ANP technique showed that “burden of special, rare, and incurable diseases” was the most important indicator in the effectiveness cluster, highlighting the role and significance of disease burden in the designation of overall health status for different groups or geographic areas. In the case of “burden of special, rare, and incurable diseases”, due to their heavy social impacts, participants have always been considered because of their responsiveness to these patients and the specific political burden that these patients can place on the system.

If a need is defined in terms of the burden of disease, morbidity, and mortality, then the lower income groups, who are presumably sicker and in greatest need, would have greater need for health services [61]. This indicator had been previously used by many other countries in resource allocation formula in the case of scarcity [16, 27, 61, 62]. The burden diseases are important indicators because the allocation of medicines to provinces that do not need it at the moment is obvious, which is result of historical distribution and ad hoc resource allocation decision-making.

According to the findings of the present study, ‘non-resident patients’ is another indicator of the efficiency cluster, which should be considered in scarce resource allocation. This finding is already confirmed by some other studies [60].

For MCDM, to gain wider use as a decision support tool, decision makers must choose techniques based on their fit to the decision context, and cognitive and time demands in collecting and analyzing their results [63]. Heretofore, despite the efforts of the Iran FDA, generally the decisions are based on emotional intelligence and there is no specifically conceptual framework or indicators to priority settings. So, although the weight of indicators has less applicability in real life, it presenting list of indicators and their priorities which should be considered in allocation decisions. Yet, there may always be disagreement about the importance of criteria when setting priorities. For this reason, ethicists have stressed the importance of fair processes, which allow key stakeholders to agree on what is legitimate and fair [26, 64]. Therefore, the accountability for reasonableness framework of Norman Daniels could further feed the allocation model proposed by this study [26]. Since efficiency, equity and effectiveness are considerations that sometimes conflict with each other, decision makers need to weigh them against each other and make trade-offs. Lastly, procedures for revising decisions in the light of reasonable challenges to them should be put in place.

### Limitations

There are some limitations that should be taken into account when interpreting the results of this study. Regarding the weighting technique choice, the fuzzy ANP is interesting from a methodological point of view but rather challenging from a pragmatic decision-making point of view (complex mathematics hard to put in action), and from an ethical point of view. ANP is an indirect method, which uses the model to prioritize and rank. Balancing the paradigms is an ethical exercise, value-laden, and which varies according to the worldview of each individual.

## Conclusions

In general, the ability to overcome inefficient resource allocation patterns caused by both oversupply and undersupply derived from historic resource allocation might be highly limited in the absence of the need indicators. The more sensible the indicators of population needs are, the more equitable distribution will be. Nevertheless, no golden rule is found regarding what weights to be. Identifying the weights of the determined indicators would necessarily be a policy decision requiring detailed considerations and discussions. Although resource allocation indicators do not reveal much difference for most provinces, their significance varies from province to province.

The development of the resource distribution models has is of great advantage for providing a structure for decision makers to set priorities in more deliberant and transparent process and also improve quality of allocation decisions through minimizing tasteful behaviors. The ANP resource allocation/distribution model developed in this study structured the scarce drug allocation problem, critical paradigms, and rationing indicators. The proposed model was superior to the previous allocation models in terms of including and quantifying interdependencies among system components. Furthermore, the application of a fuzzy theory would be effective in dealing with the intrinsic uncertainty and lack of precision posed by mapping a decision-maker’s perceptions of exact numbers. FANP approach provides IFDA with more dependable and realistic allocation scores. Both the indicator scores and the weights of the resource allocation paradigms are provided by the model. To sum up, this model is suggested to be used by decision makers in the allocation of drugs in short supply more equitably, efficiently, and effectively.

## Supplementary information

**Additional file 1.**

**Additional file 2.**

**Additional file 3.**

**Additional file 4.**

**Additional file 5.**

## Data Availability

All data is available and can be provided by the corresponding author upon rational request.

## Abbreviations

AHP: Analytic hierarchy process
ANP: Analytic network process
API: Active pharmaceutical ingredient
CI: Consistency index
CR: Consistency ratio
FANP: Fuzzy analytic network process
FLLS: Fuzzy logarithmic least squares
GP: General practitioner
IFAHP: Improved Fuzzy AHP
IFDA: Iran Food and Drug Administration
INDP: Iran National Drug Policy
MCDM: Multiple criteria decision-making
TFN: Triangular fuzzy numbers
