# Supplementary material for: An application of analytic network process model in supporting decision making to address pharmaceutical shortage
Source: BMC Health Serv Res. 2020 Jul 8;20:626. doi: 10.1186/s12913-020-05477-y (PMC7346520; doi:10.1186/s12913-020-05477-y)
Supplement: Supplementary file 1 — Additional file 1. [file 12913_2020_5477_MOESM1_ESM.docx]

Table S1: Flows of influence from each Cluster/Element*

| **Influenced by Clusters/Elements that transmit influence** | **Clusters/Elements being influenced** | | | | | | | |
| --- | --- | --- | --- | --- | --- | --- | --- | --- |
|  | **Efficiency** | | **Equity & Access** | | | **Effectiveness** | | |
|  | Total population | Non-resident patient | Number of general practitioner  and specialists | Total bed occupancy rate | Number of prescription | Burden of endemic diseases | Burden of special, rare and incurable diseases | Burden of traumatic diseases |
| **Efficiency** | 0 | | 1 | | | 1 | | |
| Total population | 0 | 0 | 1 | 1 | 1 | 1 | 1 | 1 |
| Non-resident patient | 0 | 0 | 0 | 1 | 1 | 0 | 0 | 1 |
| **Equity & Access** | 1 | | 0 | | | 0 | | |
| Number of general practitioner  and specialists | 1 | 1 | 0 | 1 | 1 | 0 | 0 | 0 |
| Total bed occupancy rate | 1 | 0 | 0 | 0 | 1 | 0 | 0 | 0 |
| Number of prescription | 0 | 0 | 0 | 0 | 0 | 0 | 0 | 0 |
| **Effectiveness** | 0 | | 1 | | | 0 | | |
| Burden of endemic diseases | 0 | 0 | 0 | 1 | 1 | 0 | 0 | 0 |
| Burden of special, rare and incurable diseases | 0 | 0 | 0 | 1 | 1 | 0 | 0 | 1 |
| Burden of traumatic diseases | 0 | 0 | 0 | 1 | 1 | 0 | 0 | 0 |

*1 shows the existence of a correlation between two elements/clusters and 0 rejects any relationship. In this Table, the numbers are ordered from row to column.
