# Supplementary material for: An application of analytic network process model in supporting decision making to address pharmaceutical shortage
Source: BMC Health Serv Res. 2020 Jul 8;20:626. doi: 10.1186/s12913-020-05477-y (PMC7346520; doi:10.1186/s12913-020-05477-y)
Supplement: Supplementary file 3 — Additional file 3. [file 12913_2020_5477_MOESM3_ESM.docx]

Table S3: The first super-matrix

|  | Efficiency | Equity and Access | Effectiveness | Goal | Total population | Non-resident patient | Number of general practitioner  and specialists | Total bed occupancy rate | Number of prescription | Burden of endemic diseases | Burden of special, rare and incurable diseases | Burden of traumatic diseases |
| --- | --- | --- | --- | --- | --- | --- | --- | --- | --- | --- | --- | --- |
| Efficiency | 0 | 1 | 0.312 | 0.166 | 1 | 1 | 0 | 0 | 0 | 0 | 0 | 0 |
| Equity and Access | 0.5 | 0 | 0.688 | 0.424 | 0 | 0 | 1 | 1 | 1 | 0 | 0 | 0 |
| Effectiveness | 0.5 | 0 | 0 | 0.409 | 0 | 0 | 0 | 0 | 0 | 1 | 1 | 1 |
| Goal | 0 | 0 | 0 | 0 | 0 | 0 | 0 | 0 | 0 | 0 | 0 | 0 |
| Total population | 0.694 | 0 | 0 | 0 | 0 | 0 | 0.147 | 0.466 | 0 | 0 | 0 | 0 |
| Non-resident patient | 0.306 | 0 | 0 | 0 | 0 | 0 | 0.123 | 0 | 0 | 0 | 0 | 0 |
| Number of general practitioner  and specialists | 0 | 0.172 | 0 | 0 | 0.059 | 0 | 0 | 0 | 0 | 0.133 | 0.113 | 0 |
| Total bed occupancy rate | 0 | 0.383 | 0 | 0 | 0.164 | 0.330 | 0.307 | 0 | 0 | 0.315 | 0.269 | 0.594 |
| Number of prescription | 0 | 0.445 | 0 | 0 | 0.140 | 0.341 | 0.423 | 0.534 | 0 | 0.552 | 0.329 | 0.406 |
| Burden of endemic diseases | 0 | 0 | 0.341 | 0 | 0.156 | 0 | 0 | 0 | 0 | 0 | 0 | 0 |
| Burden of special, rare and incurable diseases | 0 | 0 | 0.375 | 0 | 0.346 | 0 | 0 | 0 | 0 | 0 | 0 | 0 |
| Burden of traumatic diseases | 0 | 0 | 0.284 | 0 | 0.135 | 0.329 | 0 | 0 | 0 | 0 | 0.289 | 0 |
