# Supplementary material for: An application of analytic network process model in supporting decision making to address pharmaceutical shortage
Source: BMC Health Serv Res. 2020 Jul 8;20:626. doi: 10.1186/s12913-020-05477-y (PMC7346520; doi:10.1186/s12913-020-05477-y)
Supplement: Supplementary file 4 — Additional file 4. [file 12913_2020_5477_MOESM4_ESM.docx]

Table S4: The weighted super-matrix

|  | Efficiency | Equity and Access | Effectiveness | Goal | Total population | Non-resident patient | Number of general practitioner  and specialists | Total bed occupancy rate | Number of prescription | Burden of endemic diseases | Burden of special, rare and incurable diseases | Burden of traumatic diseases |
| --- | --- | --- | --- | --- | --- | --- | --- | --- | --- | --- | --- | --- |
| Efficiency | 0 | 0.5 | 0.156 | 0.166 | 0.5 | 0.5 | 0 | 0 | 0 | 0 | 0 | 0 |
| Equity and Access | 0.25 | 0 | 0.344 | 0.424 | 0 | 0 | 0.5 | 0.5 | 1 | 0 | 0 | 0 |
| Effectiveness | 0.25 | 0 | 0 | 0.409 | 0 | 0 | 0 | 0 | 0 | 0.5 | 0.5 | 0.5 |
| Goal | 0 | 0 | 0 | 0 | 0 | 0 | 0 | 0 | 0 | 0 | 0 | 0 |
| Total population | 0.347 | 0 | 0 | 0 | 0 | 0 | 0.0735 | 0.233 | 0 | 0 | 0 | 0 |
| Non-resident patient | 0.153 | 0 | 0 | 0 | 0 | 0 | 0.0615 | 0 | 0 | 0 | 0 | 0 |
| Number of general practitioner  and specialists | 0 | 0.086 | 0 | 0 | 0.0295 | 0 | 0 | 0 | 0 | 0.0665 | 0.0565 | 0 |
| Total bed occupancy rate | 0 | 0.191 | 0 | 0 | 0.082 | 0.165 | 0.1535 | 0 | 0 | 0.1575 | 0.1345 | 0.297 |
| Number of prescription | 0 | 0.2225 | 0 | 0 | 0.07 | 0.1705 | 0.211 | 0.267 | 0 | 0.276 | 0.1645 | 0.203 |
| Burden of endemic diseases | 0 | 0 | 0.1705 | 0 | 0.078 | 0 | 0 | 0 | 0 | 0 | 0 | 0 |
| Burden of special, rare and incurable diseases | 0 | 0 | 0.1875 | 0 | 0.173 | 0 | 0 | 0 | 0 | 0 | 0 | 0 |
| Burden of traumatic diseases | 0 | 0 | 0.142 | 0 | 0.0675 | 0.1645 | 0 | 0 | 0 | 0 | 0.1445 | 0 |
