# Supplementary material for: An application of analytic network process model in supporting decision making to address pharmaceutical shortage
Source: BMC Health Serv Res. 2020 Jul 8;20:626. doi: 10.1186/s12913-020-05477-y (PMC7346520; doi:10.1186/s12913-020-05477-y)
Supplement: Supplementary file 5 — Additional file 5. [file 12913_2020_5477_MOESM5_ESM.docx]

Table S5: The limit super-matrix

|  | Efficiency | Equity and Access | Effectiveness | Goal | Total population | Non-resident patient | Number of general practitioner  and specialists | Total bed occupancy rate | Number of prescription | Burden of endemic diseases | Burden of special, rare and incurable diseases | Burden of traumatic diseases |
| --- | --- | --- | --- | --- | --- | --- | --- | --- | --- | --- | --- | --- |
| Efficiency | 0.206 | 0.206 | 0.206 | 0.206 | 0.206 | 0.206 | 0.206 | 0.206 | 0.206 | 0.206 | 0.206 | 0.206 |
| Equity and Access | 0.256 | 0.256 | 0.256 | 0.256 | 0.256 | 0.256 | 0.256 | 0.256 | 0.256 | 0.256 | 0.256 | 0.256 |
| Effectiveness | 0.096 | 0.096 | 0.096 | 0.096 | 0.096 | 0.096 | 0.096 | 0.096 | 0.096 | 0.096 | 0.096 | 0.096 |
| Goal | 0.000 | 0.000 | 0.000 | 0.000 | 0.000 | 0.000 | 0.000 | 0.000 | 0.000 | 0.000 | 0.000 | 0.000 |
| Total population | 0.093 | 0.093 | 0.093 | 0.093 | 0.093 | 0.093 | 0.093 | 0.093 | 0.093 | 0.093 | 0.093 | 0.093 |
| Non-resident patient | 0.033 | 0.033 | 0.033 | 0.033 | 0.033 | 0.033 | 0.033 | 0.033 | 0.033 | 0.033 | 0.033 | 0.033 |
| Number of general practitioner  and specialists | 0.028 | 0.028 | 0.028 | 0.028 | 0.028 | 0.028 | 0.028 | 0.028 | 0.028 | 0.028 | 0.028 | 0.028 |
| Total bed occupancy rate | 0.084 | 0.084 | 0.084 | 0.084 | 0.084 | 0.084 | 0.084 | 0.084 | 0.084 | 0.084 | 0.084 | 0.084 |
| Number of prescription | 0.116 | 0.116 | 0.116 | 0.116 | 0.116 | 0.116 | 0.116 | 0.116 | 0.116 | 0.116 | 0.116 | 0.116 |
| Burden of endemic diseases | 0.024 | 0.024 | 0.024 | 0.024 | 0.024 | 0.024 | 0.024 | 0.024 | 0.024 | 0.024 | 0.024 | 0.024 |
| Burden of special, rare and incurable diseases | 0.034 | 0.034 | 0.034 | 0.034 | 0.034 | 0.034 | 0.034 | 0.034 | 0.034 | 0.034 | 0.034 | 0.034 |
| Burden of traumatic diseases | 0.030 | 0.030 | 0.030 | 0.030 | 0.030 | 0.030 | 0.030 | 0.030 | 0.030 | 0.030 | 0.030 | 0.030 |
